# Laboratory Investigations on the Diagnosis of Tuberculosis in the Malnourished Tribal Population of Melghat, India

**DOI:** 10.1371/journal.pone.0074652

**Published:** 2013-09-12

**Authors:** Rajpal S. Kashyap, Amit R. Nayak, Hari M. Gaherwar, Shraddha S. Bhullar, Aliabbas A. Husain, Seema D. Shekhawat, Ruchika K. Jain, Sonali S. Gaikwad, Ashish R. Satav, Hemant J. Purohit, Girdhar M. Taori, Hatim F. Daginawala

**Affiliations:** 1 Biochemistry Research Laboratory, Central India Institute of Medical Sciences, Nagpur, Maharashtra, India; 2 Meditation, AIDS, Health, Addiction & Nutrition (MAHAN) Trust, C/O Mahatma Gandhi Tribal Hospital, Karmagram, Utavali, Tahsil Dharni, Amaravati, Maharashtra, India; 3 Environmental Genomics Unit, National Environmental Engineering Research Institute,Nagpur, Maharashtra, India; St. Jude Children's Research Hospital, United States of America

## Abstract

**Background:**

Malnutrition is a major risk factor for the development of tuberculosis (TB). In India, Melghat is among the tribal regions which consist of highest number of malnutrition cases. Because of the paucity of TB data from these malnourished areas there is an urgent need for the development and evaluation of improved TB diagnostic tests. In the present study, three in house developed diagnostic tests namely TB-Ag(antigen) ELISA, Adenosine deaminase (ADA) estimation and IS*6110* polymerase chain reaction (PCR) assay were investigated for the detection of *Mycobacterium tuberculosis* (*M. tb.*) infection.

**Methods:**

For investigation, blood samples were collected from 128 study subjects from six villages of Melghat tribal area and evaluated using three in house developed assays, namely TB-Ag ELISA, ADA estimation and IS*6110* PCR.

**Results:**

The TB-Ag ELISA method yielded 83% sensitivity and 94% specificity. The ADA and PCR assay gave a sensitivity of 61% and 49% and specificity of 62% and 98% respectively. A considerable good agreement of 82.81% (k=0.472) between TB-Ag ELISA and PCR was observed. The overall sensitivity of TB-Ag ELISA was significantly higher (p<0.05) than the ADA and PCR while PCR yielded highest specificity among all the three evaluated tests.

**Conclusions:**

We concluded that the routine use of TB-Ag ELISA can be useful for screening of suspected TB patients in the malnourished population where sophisticated laboratory set up is difficult.

## Introduction

Tuberculosis (TB) is a major health problem worldwide. As per the World Health Organization (WHO) report of 2010, India is the highest TB burden country with an estimated TB incidence figure of 2.3 million cases out of a global 9.4 million cases. The WHO statistics also showed that India is 17^th^ out of the 22 high burden countries in terms of TB incidence rate [1]. It is estimated that about 40-50% of the Indian population is infected with TB bacteria, the vast majority of whom have latent rather than active TB. The association between TB and malnutrition is well recognized; TB can lead to malnutrition and malnutrition may predispose to TB [2]. TB has been found to coexist with malnutrition among patients at the time of starting treatment in both developed and developing countries [3]. TB is also associated with various socioeconomic factors and often occurs in populations suffering from poverty, poor housing and economic deprivation and these are also the major factors predisposing to poor nutritional status and impaired immune function [4].

Melghat is a hilly forest tribal area in Maharashtra State of India with a tribal population of nearly 0.28 million. Nearly 80% of the population of this region suffers from malnutrition. Most of the villagers (>90%) are farmers or labourers, with below poverty line status (>75%) and living in kaccha houses without electricity (>90%) and having high illiteracy rates (>50%). In Melghat, our investigation team has observed that TB is one of the most important and leading cause of death in the economically productive age group of 16-40 years. However, to the best of our knowledge, data of TB incidence have not been yet documented in this tribal region.

Earlier in our laboratory, we have developed ELISA based TB antigen (Ag) detection test based on the evaluation of Ag 85 complex in the body fluid of TB patients [5,6]. Adenosine deaminase (ADA) has also been extensively evaluated in the body fluids of pulmonary TB (PTB) and extra pulmonary TB (EPTB) patients with reliable sensitivity and specificity [7]. Both the developed methods are inexpensive, relatively simple, and can be performed in any pathology laboratory where sophisticated tests are not available. In addition to that, an in house *IS6110* based Polymerase chain reaction (PCR) assay was also developed in our laboratory to detect *Mycobacterium tuberculosis* (*M. tb.*) DNA in cerebrospinal fluid (CSF) samples of Tuberculous meningitis (TBM) patients [8]. Because of the paucity of TB data from this malnourished area, there is a need for evaluating TB diagnostic tests for the rapid diagnosis of the patients in such remote region. The objective of the present study is to evaluate the performance of above mentioned three tests TB-Ag ELISA, ADA and PCR in the malnourished tribal population to estimate the burden of TB in Melghat region.

## Materials and Methods

### Ethics statement

The study was approved by Ethical Committee of Central India Institute of Medical Sciences (CIIMS), Nagpur and Meditation, Aids, Health Addiction and nutrition (MAHAN) Trust, Melghat. Written consents were taken from each participant after detailed oral explanation about the study. Since the study was also conducted on children, a written informed consent was obtained from the immediate caretaker, or next of kin, prior to inclusion, on behalf of children participating in the study.

### Study design

The present study was conducted in high burden malnutrition tribal region of Melghat villages, Amravati district, India. The region has high prevalence of undiagnosed TB along with other infectious diseases. The participants recruited were mostly tribal’s form six different villages of Melghat most of which had poor infrastructure development and socioeconomic status, no provisions of electricity, toiletries and ate nutrients deficient diets. Each participant was asked to complete a questionnaire about his/her possible risk of exposure to *M. tb*. Details of any prior Tuberculin skin test (TST), presence of underlying illnesses, infections experienced in the last 3 months, medication, history of previous TB or anti-TB treatment and daily dietary intake were also recorded. Baseline characteristics such as age, gender, weight, height, type and duration of exposure with active TB patients, occupation, and education were recorded. Bacillus Calmette–Guérin (BCG) vaccination status was assessed based on examination of BCG scar on left forearm.

### Samples

Blood samples (n=128) were collected in plain tube and on FTA card for TB-Ag ELISA, ADA test and for **the** DNA isolation followed by PCR amplification of *M. tb*. specific gene *IS6110*. **The** collected samples were stored at -20°C or at room temperature until further analysis.

### Subjects

Study populations were classified into following two categories on the basis of their clinical **characteristics.**

***ActiveTBsubjects* (*n=41***)**: TB (n=37**)** was confirmed if acid fast bacilli (AFB**)** and/or culture of sputum specimens were positive for *M.tb*. When both tests were negative (n=04**)**, the patients were diagnosed based on clinical symptoms**. Clinical suspicion of TB was based on minimum of 3 of the following symptoms a) Chronic cough with or without expectoration/hemoptysis/chest pain of more than 2–3 weeks or past history of TB b) Fever more than 2–3 weeks c) Progressive unexplained weight loss d) loss of appetite e) night sweats f) or by chest radiographs g) response to anti TB treatment (ATT).

***Non-TBcontrols* (*n=87***): This category included participants from the same villages. All the control cases included in this group were TST, sputum and culture negative with no clinical, bacteriological features of TB, and normal chest radiograph with no history of ATT.

### TB Antigen ELISA test

*M. tb*. specific antigen (Ag85) in the serum samples were evaluated by the indirect ELISA protocol as described earlier by Kashyap et al. [5]. Negative reference control was selected from the pooled serum of non-TB healthy controls that had never been exposed to TB, and the absence of Ag 85 complex antigen was demonstrated by immunoblotting with specific rabbit antibodies against Ag 85 complex antigen [6]. Positive reference control Ag85 complex from M. *tb* strain H _37_R_v_ was obtained from Colorado State University, USA through TB Research Materials and Vaccine Testing Contract (NO1- AI-40091) [5]. A single dilution (10µg/ml) of the positive stock was made in the negative reference serum to achieve a defined reactivity, which was within the accurate detection limits of the ELISA. ELISA was run with the positive serum control, negative serum control and sample blank (PBS). Three replicates of positive and negative controls were included on each ELISA plate along with the test sample. A sample with an absorbance of >0.18 was considered positive for ELISA result as previously reported [6]. The accuracy of test was evaluated by determining its sensitivity and specificity as compared to reference standards**.**

### ADA

ADA activity in serum samples was determined by the method of Guisti and Galanti [9] based on the Berthlot reaction, which is the formation of coloured indophenol complex from ammonia liberated during deamination of adenosine. The development of colour was quantified by spectrophotometer (Systronic, India). One unit of ADA is defined as the amount of enzyme required to release 1mMol of ammonia/min from adenosine at standard assay conditions. Results were expressed as units per liter per minute (U/L/min). A sample with cut-off value of ≥15 U/L/min was considered as a positive test result.

### DNA isolation

Sample collection and DNA isolation was done by FTA Elute Technology. 100 µl of whole blood samples obtained by venous puncture were applied to FTA Elute matrix. The samples were dried thoroughly and kept at room temperature until use. For isolating the DNA, a 3 mm disc was punched from the cards containing the samples and placed in a microcentrifuge tube. The punch was rinsed in 500 µl water and pulse vortexed three times for 5 seconds. Water was removed and tube was centrifuged for 5 seconds. 30µl sterile water was added to it, sample was heated at 95°C for 60 min; pulse vortexed 30 times; and centrifuged at 12000 rpm for 10 min. 10 µl of eluted DNA was used further for PCR.

### IS6110 gene PCR

Identification of *M. tb*. was done using a specific pair of primers designed to amplify an insertion sequence IS*6110* in the *M. tb*. complex with the expected band size of about123-bp. The sequence of these primers are: 5' CCT GCG AGC GTA GGC GTC GG 3' and 5' CTC GTC CAG CGC CGC TTC GG 3' respectively. A 50 µl reaction contained 10× assay buffer (Applied Biosystems, USA), 10 mM dNTP’s (Applied Biosystems, USA), 10 pmole of each primer (Sigma Aldrich, USA), 2.5 units Taq DNA Polymerase (Applied Biosystems, USA) and 5 µl of extracted DNA. Amplification was carried out in a thermal minicycler (peqlab Biotechnologie GmbH, Erlangen, Germany), which involved 40 cycles of denaturation at 94°C for 1 min, annealing of primers at 68°C for 1 min, and primer extension at 72°C for 1 min. In each independent PCR assay, test results were compared with the results for one positive and one negative control. The positive controls included the DNA of H_37_Rv strain provided by Colorado State University, USA, (Contract No 1-A1-40091). No-template control reaction consisted of sterile water instead of target template. The amplification products were separated on 2% agarose gels, visualized on a UV- light transilluminator (Biotech R & D Laboratories, Yercaud, Salem, India) and photographed. The test samples were analyzed on the basis of presence or absence of 123 bp product as compared to the positive control DNA.

### Statistical analysis

Statistical analysis was performed using the MedCalc statistical software (version 10.0.1). Optimal cut-off value for the ADA was calculated by using ROC analysis, and enzyme activity of 15 U/L/min was set as cut-off for the TB patient in a serum sample. Baseline characteristic between non-TB controls and TB patients were compared using the test for proportion. Association of positivity and the studied risk factor in both active and non-TB control group were measured using chi-square test. Similarly the sensitivity and specificity of three tests were compared with each other using the Chi-square test. Agreements between the tests were calculated with the help of κ- Statistics (McNemar’s test) and the strength of the agreement were reported in terms of kappa (κ) value. *P*-Value <0.05 were considered as statistically significant for all the tests.

## Results

During the course of **the** study period, 128 participants were enrolled for the study. TB was diagnosed in 41 (32%) participants out of whom 21 were males and 20 were females, with the mean age of 34.02 years (range 17-65 years). **Among active TB group, 90% (37/41**)** were positive for acid fast bacilli (AFB**)** and/or *M.tb* sputum culture while TB in 10% (4/41**)** participants was diagnosed on basis of clinical symptoms**. Remaining 87 (68%) participants were categorized into non-TB control group which included 51 males and 36 females, with a mean age of 35.06 years (range 10-70 years). All **the** participants recruited in the study were negative for HIV infection, and were not receiving any immunosuppressive drugs. The baseline characteristics of active TB and non-TB control population are shown in Table 1. There was significantly (p < 0.05) more number of patients with weight loss, cough, smoking and tobacco habits in active TB group as compared to non-TB control group. Similarly, we found patients with low body mass index (BMI) more (p < 0.05) in active TB group than in non-TB control population.

**Table 1:** The baseline characteristics of this active TB and non-TB control population.

**Characteristics**	**Active TB**	**Non-TB Control**	***P-value***
	**(n=41**)	**(n=87**)	
Age			
*<18*	1 (2)	9 (10)	0.2118
*18 to 40*	32 (78)	53 (61)	0.0891
*>40*	8 (20)	25 (29)	0.3865
Gender			
*Male*	21 (51)	50 (57)	0.6553
*Female*	20 (49)	37 (43)	0.6553
BCG Vaccinated			
*Yes*	12 (29)	23 (26)	0.8859
*Don’t Know*	10 (24)	17 (20)	0.776
BMI			
**Underweight (16-18.5**)	18 (44)	22 (25)	0.0497
**Normal (18.5 to 25**)	19 (46)	60 (69)	0.0213
**Overweight (> 25**)	4 (10)	5 (6)	0.654
Weight loss	18 (44)	16 (18)	0.0037
Cough with Expectorant	**20 (49)**	**17 (20)**	**0.0016**
Cough without Expectorant	**21 (51)**	**70 (80)**	**0.0016**
Fever	18 (44)	25 (29)	0.1406
Abdominal pain	14 (34)	27 (31)	0.8914
Chest pain	9 (22)	9 (10)	0.1188
Smoking	4 (10)	1 (1)	0.0489
Alcohol	6 (15)	10 (11)	0.7228
Tobacco	15 (37)	8 (9)	0.0003

Note: n= no of cases; ( ) = Percentage

Table-**2** Shows results of the TB-Ag ELISA in TB and non-TB control group. The age wise positivity of TB-Ag ELISA was around 100% (1/1), 81% (26/32), 88% (7/8) and 0%, 5% (3/53) and 8% (2/25) respectively among three age groups (i.e <18, 18-40 and >40), in TB and non-TB control group. Test positivity among the BCG vaccinated individual was found to be 100% in active TB group, interestingly very low positivity (4%) was observed in non-TB control group. Similarly among underweight BMI category positivity was around 89% (16/18) and 4% (1/22) respectively in TB and non-TB control group.

**Table 2:** TB-Ag ELISA assay.

**Characteristics**	**n=41**	**Active TB**	**Active TB**	**Chi-square**	**Df**	***P*-value**	**n=84**	**Non-TB Control**	**Non-TB Control**	**Chi-square**	**Df**	***P*-value**
		**Positive**	**Negative**					**Positive**	**Negative**			
		**n= 34 (83%**)	**n= 7 (17%**)					**n= 5 (6%**)	**n= 82 (94%**)			
**Age**												
<18	1	1 (100)	0				9	0	9 (100)			
18 to 40	32	26 (81)	6 (19)				53	3 (5)	50 (95)			
>40	8	7 (88)	1 (12)	30.059	2	**0.0001**	25	2 (8)	23 (92)	0.000	1	1.0000
**Gender**												
Male	21	16 (76)	5 (24)				50	3 (6)	47 (94)			
Female	20	18 (90)	2 (10)	0.029	1	0.8638	37	2 (5)	35 (95)	0.000	1	1.0000
**BCG Vaccinated**												
Yes	12	12 (100)	0				23	1 (4)	22 (96)			
No	19	14 (74)	5 (26)				47	2 (4)	45 (96)			
Don’t Know	10	8 (80)	2 (20)	1.647	2	0.4389	17	2 (12)	15 (88)	0.400	2	0.8187
**BMI**												
**Underweight (16-18.5**)	18	16 (89)	2 (11)				22	1 (4)	21 (96)			
**Normal (18.5 to 25**)	19	14 (74)	5 (26)				60	3 (5)	57 (95)			
**Overweight (> 25**)	4	4 (100)	0	7.294	2	**0.0261**	5	1 (20)	4 (80)	1.600	2	0.4493

Depict the results of the M. tuberculosis Ag85 estimation in the serum of TB patients and non-TB control individuals in Melghat region. Note: n = no of cases; ( ) = Percentage; BMI = Body Mass Index; Ag85 = Antigen 85.

Table 3 Shows the results of ADA assay among the TB and non-TB control groups. The overall positivity for ADA assay was around 61% (25/41) in the active TB group and 38% (33/87) in non-TB control group. In active TB group, 72% (23/32) of participants in age group of 18-40 were ADA positive whereas 36% (19/53) were positive in non TB control group. The participants in underweight category of active TB group showed 56% (10/18) ADA positivity. However, in the same category of non-TB control group, 27% (6/22) positivity for ADA activity was observed.

**Table 3:** ADA assay.

**Characteristics**	**n=41**	**Active TB**	**Active TB**	**Chi-square**	**Df**	***P*-value**	**n=84**	**Non-TB Control**	**Non-TB Control**	**Chi-square**	**Df**	***P*-value**
		**Positive**	**Negative**					**Positive**	**Negative**			
		**n= 25 (61%**)	**n= 16 (39%**)					**n= 33 (38%**)	**n= 54 (62%**)			
**Age**												
<18	1	0 (0)	1 (100)				9	6 (67)	3 (33)			
18 to 40	32	23 (72)	9 (28)				53	19 (36)	34 (64)			
>40	8	2 (25)	6 (75)	**16.000**	**1**	**0.0001**	25	8 (32)	17 (68)	**8.909**	**2**	**0.0116**
**Gender**												
Male	21	12 (57)	9 (43)				50	21 (42)	29 (58)			
Female	20	13 (65)	7 (35)	0.000	1	1.0000	37	12 (32)	25 (68)	1.939	1	0.1637
**BCG Vaccinated**												
Yes	12	5 (42)	7 (58)				23	9 (39)	14 (61)			
No	19	13 (68)	6 (32)				47	19 (40)	28 (60)			
Don’t Know	10	7 (70)	3 (30)	4.160	2	0.1249	17	5 (29)	12 (71)	**9.455**	**2**	**0.0089**
**BMI**												
**Underweight (16-18.5**)	18	10 (56)	8 (44)				22	6 (27)	16 (73)			
**Normal (18.5 to 25**)	19	13 (68)	6 (32)				60	27 (45)	33 (55)			
**Overweight (> 25**)	4	2 (50)	2 (50)	**7.760**	**2**	**0.0207**	5	0 (0)	5 (100)	**12.121**	**1**	**0.0005**

Depict the results of ADA assay in the serum of TB patients and non-TB control individuals in Melghat region. Note: n = no of cases; ( ) = Percentage; BMI = Body Mass Index; ADA = Adenosine Deaminase

Table **4** shows** the results of the *IS6110* PCR assay in the TB and non TB blood samples. The *IS6110* PCR assay was positive in 49% (20/41**)** of TB cases and 2% (2/87**)** in non-TB control cases. The majority of the cases under the study population were in the age group of 18-40 years yielding 53% (17/32**)** positivity in active TB cases**. The BMI of majority of individuals were under the categories of underweight, normal in both the PCR positive and PCR negative cases of the active TB group. The BCG vaccination status was less in both the groups of active and non-TB control group.

**Table 4:** PCR assay.

**Characteristics**	**n=41**	**Active TB**	**Active TB**	**Chi-square**	**Df**	***P*-value**	**n=84**	**Non-TB Control**	**Non-TB Control**	**Chi-square**	**Df**	***P*-value**
		**Positive**	**Negative**					**Positive**	**Negative**			
		**n=20 (49%**)	**n=21 (51%**)					**n=2 (2%**)	**n=85 (98%**)			
**Age**												
<18	1	0	1 (100)				9	0	9 (100)			
18 to 40	32	17 (53)	15 (47)				53	2 (4)	51 (96)			
>40	8	3 (38)	5 (62)	**8.450**	**1**	**0.0037**	25	0	25 (100)			
**Gender**												
Male	21	9 (43)	12 (57)				50	0	50 (100)			
Female	20	11 (55)	9 (45)	0.050	1	0.8231	37	2 (5)	35 (95)			
**BCG Vaccinated**												
Yes	12	4 (33)	8 (67)				23	0	23 (100)			
No	19	12 (63)	7 (37)				47	1 (2)	46 (98)			
Don’t Know	10	4 (40)	6 (60)	**6.400**	**2**	**0.0408**	17	1 (6)	16 (94)	0.500	1	0.4795
**BMI**												
**Underweight (16-18.5**)	18	8 (44)	10 (56)				22	1 (5)	21 (95)			
**Normal (18.5 to 25**)	19	10 (53)	9 (47)				60	1 (2)	59 (98)			
**Overweight (> 25**)	4	2 (50)	2 (50)	**5.200**	**2**	**0.0743**	5	0	5 (100)	0.500	1	0.4795

Depict the results of Mycobacterium specific IS6110 (123bp) PCR product in the blood of TB patients and non-TB control individuals in Melghat region. Note: n = no of cases; ( ) = Percentage; BMI = Body Mass Index; PCR = Polymerase Chain Reaction

Based on the above results of Ag85 ELISA, ADA and PCR, we found that baseline characteristics, such as age and **BMI were found to be associated with the Ag85 (P < 0.05**)** and PCR (P<0.05 only for age**)** positivity in active TB group** (Tables **2** and **4**)**. While ADA was found to be significantly (P<0.05**)** associated with age, sex and BMI levels in both active and control group (** Table **3**)**.**

Table **5** depicts **the performance** of TB-Ag ELISA, PCR assay and ADA activity in TB and non-TB control patients. Mean absorbance of Ag85 ELISA in TB group was, 0.312±0.092 which was **significantly (p<0.05**)** higher than non-TB control group (0.165±0.064**) (Figure **S2**). Similarly, positivity of PCR assay in TB patients was around 48.78% which was found to be significant (p < 0.0001) than control group (2.30%). Mean ADA activities in TB and non-TB control groups were 15.846±4.70 and 14.279±4.22, respectively. No significant difference in ADA activity between active TB and non-TB control group was observed.

**Table 5:** Performance of TB-Ag ELISA, ADA, and PCR assay in active TB and non-TB control population.

**Test**	**Non-TB Control (n=87**)	**Active TB (n=41**)	**95%CI**	***p-value***
TB Ag ELISA (Absorbance)	0.165±0.064	0.312±0.092	0.1192 to 0.1748	p < 0.0001
ADA Test (Enzyme activity)	14.279±4.22	15.846±4.70	-0.0743 to 3.2083	p = 0.0611
PCR (%positivity)	2.30%	48.78%	30.875% to 61.309%	p < 0.0001

The overall sensitivity and specificity of TB-Ag ELISA, ADA & PCR were 83%, 61% 49% and 94%, 62% & 98% respectively (as shown in table 6). Sensitivity of Ag85 ELISA was found to be significantly high (p<0.05) as compared to ADA and PCR. Similarly, based on the comparison of specificities of these tests we found that the specificity of Ag85 ELISA and PCR were considerably high (p<0.05) as compared to ADA.

**Table 6:** Overall sensitivity and specificity of assays.

	**TB-Ag ELISA**	**ADA**	**PCR**
Sensitivity	83% (34/41)^*#^	61% (25/41)	49% (20/41)
Specificity	94% (82/87)^*^	62% (54/87)	98% (85/87)^@^

Overall sensitivity and specificity of TB-Ag ELISA, ADA and PCR tests in the Melghat region. Note: *p <0.05 and ^@^ *p* <0.05 and with respect to ADA; similarly ^#^ *p* <0.05 with respect to PCR

Table 7 depicts **the** results of concordance analysis, between TB-Ag ELISA, ADA and PCR tests for 128 patients. Considerable agreement of 82.81% (κ = 0.472; 95% CI, 0.272-0.673) was observed between Ag85 ELISA and PCR test among the studied population. While a poor agreement was observed between ADA and Ag85 ELISA i.e. 57.81% (κ = 0.111; 95% CI, -0.0681-0.29) and also between ADA and PCR 54.68% (κ = 0.0343; 95% CI, -0.149 to 0.218).

**Table 7:** Concordance between three assays.

	**Ag85 Positive (n=30**)	**Ag85 Negative (n=98**)	**Concordance**	**κ (95% CI**)
ADA Positive (n=58)	17	41	57.81	0.111 (-0.0681-0.292)
ADA Negative (n=70)	13	57		
**PCR Positive (n=22**)	15	7	82.81	0.472 (0.272-0.673)
**PCR Negative (n=106**)	15	91		
	**PCR Positive (n=22**)	**PCR Negative (n=106**)		
ADA Positive (n=25)	11	47	54.68	0.0343 (-0.149 to 0.218)
ADA Negative (n=16)	11	59		

Concordance between TB-Ag ELISA, ADA, PCR test for TB in the Melghat population (n=128). Note: κ = Kappa value; 95% CI = 95% Confidence interval

## Discussion

The tribal population of Melghat received considerable attention in recent past due to the higher percentage of mortality reported among children due to malnutrition [10]. The association between TB and malnourishment is well recognized. Various socioeconomic factors like poverty, high crowding index area, economic deprivation and poor nutrition are often associated with TB. The increasing risks due to spread of TB among the studied tribal population have made early diagnosis a matter of utmost concern. Analysis of baseline data in the present study population indicated significant association of risk factors, such as BMI, weight loss, cough, fever, smoking and socio-economic status with active TB group than control population. Development of newer and more efficient diagnostic tests that can identify TB cases with better specificity and sensitivity are thus urgently needed in this region.

In the recent years, many *M. tb*. antigens were used for rapid diagnosis of TB [11,12,13]. Ag85 complex of *M.tb* is among one such antigen which has been explored by many researchers for TB diagnosis in the last two decades [13,14,15,16]. We have previously demonstrated the presence of an Ag85 complex in the TBM and TB patients and reported the sensitivity and specificity of more than 80% [5,6]. Encouraging results were obtained when the same assay was performed in the clinical samples of the tribal population of Melghat. A significant difference was observed in mean absorbance between active TB and control group. The observed sensitivity and specificity for Ag85 ELISA was 83% and 94%, respectively which is reasonably good. Similarly, the risk factors such as age and BMI were also significantly associated with Ag85 positivity in active TB group compared to control. Although many reports suggest that Ag85 complex may show cross-reactivity in BCG vaccinated population as it is present in both *M. bovis* and *M. tb*. strain [16,17,18], however we have not seen any impact of BCG vaccine in the diagnosis of TB using Ag85 complex. Our results for BCG and non-BCG vaccinated population, indicate that in spite of homology between Ag85 and BCG, Ag85 complex can be effectively used in TB diagnosis in this region.

ADA is basically an essential enzyme involved in the metabolism of purine nucleosides and considered as a reliable marker of cell-mediated immunity. High ADA levels in pleural, serum and CSF samples have been reported in PTB and EPTB [7,19,20]. Due to its simplicity and low cost, it is suggested as a useful tool in the area with high TB incidence rates and limited resources. The sensitivity and specificity of ADA assay observed in this region was 61% and 62% respectively, which is less than TB-Ag ELISA. No significant difference in ADA activity was observed between active and control group. As mentioned, ADA is a marker of activated T cells; hence it is obvious that it may be elevated in other infections also [19]. As already discussed earlier, these population lives in areas, with poor access to the health and majority of the population is consuming nutrient deprived meals which generally lead to malnutrition among them. Other factors such as poor hygienic practices, lack of awareness of diseases and available services, deficiency of certain vitamins and minerals like vitamin A, B complex, C, E and selenium, which are fundamental to the integrity of the immune response, are prevalent among the studied population [21,22,23]. It is expected that the present population might be having infections other than TB, due to which increased ADA levels were observed in certain participants. Inspite of reported literature regarding the TB diagnostic potential of ADA test, less sensitivity was observed in our studied population which suggests that ADA test is not suitable for TB identification atleast in this region.

Another test used in the present study was PCR which is highly sensitive method for the detection of *M. tb*. An in-house PCR method using a specific pair of primers designed to amplify the insertion sequence, IS*6110* in the *M. tb*. genome has been previously reported by us to analyze CSF [8]. PCR in blood samples has been proved to be a rapid and specific technique but sensitivity has proved to be lower [24,25]. We evaluated the sensitivity and specificity of a PCR system for the detection of *M. tb*. in blood samples spotted on FTA cards. The assay was positive in only 49% (20/41) of the active TB cases. However, the risk factors, such as age, BMI and BCG vaccination status were significantly associated with PCR positivity in active TB group than the control group. On similar lines, % positivity of PCR assay in TB group was more significant than control group. Even though in our earlier study, we obtained a very good results with existing PCR system and the assay was found to be as sensitive and specific showing good correlation with the in-house TB-Ag ELISA technique [26], however, we did not have satisfactory findings in the present study. The reason could be due to the use of FTA card for DNA isolation procedure. This is the only difference we have from our previous protocols, where isolation of DNA was by phenol chloroform isoamyl or Chelex methods [27]. The performance of PCR in blood collected on FTA card has not been reported for the molecular diagnosis of TB. Secondly, it might be possible that during transportation and handling, there are many chances of contamination. There is a need for refinement of this assay so that it can be effectively used in such regions.

Based on sensitivity and specificity of TB-Ag ELISA test observed in our study, it can be concluded that the present test is reliable in diagnosis of TB in tribal population of Melghat. Since TB-Ag ELISA is a quantitative test, it can be used for follow up of the patients. In few cases, TB-Ag ELISA detect antigen, when no clinical history of TB has been reported. One cannot rule out the possibility of latent TB infection, as this test may detect antigen in predormal or latent stages of TB infection. In such cases, the detailed history of patients should be recorded and recommendation of repeat testing after interval of 3-4 months up to one year might be useful. Our earlier experiences (our unpublished observation) have shown that in some Ag positive cases, where no clinical sign of TB is reported, patients developed TB symptoms after a period of time (3 to 12 months). Under these conditions, it is recommended to repeat the test at regular intervals and correlate clinically.

In conclusion, TB-Ag ELISA is better than ADA and PCR and can be used as a potential diagnostic test for screening of patient for TB infection in the **tribal population** of Melghat where the sophisticated laboratory set up is not available. The results provide vital information on the TB disease situation amongst this population and can serve as baseline data for future evaluation of the impact of disease control measures and epidemiological trends.

## Supporting Information

Figure S1 **TB Antigen ELISA test.**
(DOC)

Figure S2 **Scattered plot of TB Ag ELISA in TB (n=41) and non TB control (n=87) patients.**
(JPG)
