# Supplementary material for: Laboratory Investigations on the Diagnosis of Tuberculosis in the Malnourished Tribal Population of Melghat, India
Source: PLoS One. 2013 Sep 12;8(9):e74652. doi: 10.1371/journal.pone.0074652 (PMC3772098; doi:10.1371/journal.pone.0074652)
Supplement: Figure S1 — TB Antigen ELISA test. (DOC) [file pone.0074652.s001.doc]

**S1**

**TB Antigen ELISA test**

*M. tb.* specific antigen (Ag85) in the serum samples were evaluated by the indirect ELISA protocol as described earlier by Kashyap *et al.* [5]. 100µl of serum sample (1:200 diluted in PBS) were coated on the wells of microtiter plates and incubated for 90 min at 37°C. After incubation wells were then washed once with Phosphate buffer saline Tween20 (PBST) and blocked with blocking buffer (0.5% BSA in PBST). After blocking, wells were washed three times with PBST followed by addition of 100µl monoclonal antibody (1:2,000 dilutions in PBST) against TB antigen (University of Colorado, USA). After 45 min of incubation the wells were washed three time with the PBST and 100µl secondary antibody, affinity purified goat anti-rabbit IgG (1:10000 dilution in PBST) conjugated to horseradish peroxidise (Genei, Bangalore, India)was added to wells and incubated at 37°C for 45 min. After incubation the wells were washed four times extensively with PBST followed by addition of 100µl of TMB/H2O2 substrate and incubated at room temperature for 10 min. The reaction was stopped with addition of 100µl of 2.5 N H2SO4 and the absorbance of colour in each well was read at 450 nm. Negative reference control was selected from the pooled serum of non-TB healthy controls that had never been exposed to TB, and the absence of Ag 85 complex antigen was demonstrated by immunoblotting with specific rabbit antibodies against Ag 85 complex antigen [6]. Positive reference control Ag85 complex from M. *tb* strain H_37_R_v_ was obtained from Colorado State University, USA through TB Research Materials and Vaccine Testing Contract (NO1- AI-40091) [5]. A single dilution (10µg/ml) of the positive stock was made in the negative reference serum to achieve a defined reactivity, which was within the accurate detection limits of the ELISA. ELISA was run with the positive serum control, negative serum control and sample blank (PBS). Three replicates of positive and negative controls were included on each ELISA plate along with the test sample. A sample with an absorbance of >0.18 was considered positive for ELISA result as previously reported [6]. The accuracy of test was evaluated by determining its sensitivity and specificity as compared to reference standards**.**
