# Supplementary figures and images for: Laboratory Investigations on the Diagnosis of Tuberculosis in the Malnourished Tribal Population of Melghat, India
Source: PLoS One. 2013 Sep 12;8(9):e74652. doi: 10.1371/journal.pone.0074652 (PMC3772098; doi:10.1371/journal.pone.0074652)

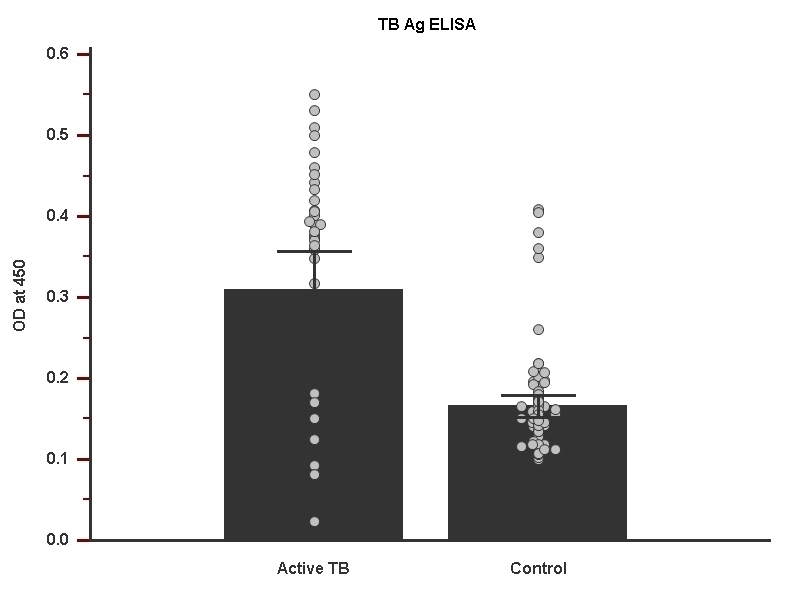

Supplement: Figure S2 — Scattered plot of TB Ag ELISA in TB (n=41) and non TB control (n=87) patients. (JPG) [file pone.0074652.s002.jpg]
